# Physical Characteristics of Elite Male Bandy Players

**DOI:** 10.3390/ijerph191912337

**Published:** 2022-09-28

**Authors:** Henrik Petré, Alexander Ovendal, Niklas Westblad, Lasse Ten Siethoff, Niklas Psilander

**Affiliations:** Department of Physiology, Nutrition and Biomechanics, The Swedish School of Sport and Health Sciences, 114 33 Stockholm, Sweden

**Keywords:** elite athletes, testing, team sports, physical profile

## Abstract

Physical characteristics of elite male bandy players have not been studied for the last 30 years. Therefore, the purpose of this study was to evaluate the physical characteristics of elite male bandy players with respect to playing positions. A cross-sectional study was performed that included 25 male bandy players from one of the highest-ranked bandy leagues in the world. Body weight, length, isometric mid-thigh pull, countermovement jump, squat jump, unilateral long jump, bilateral long jump, 15- and 30-m sprint, 15-m flying sprint, and VO_2max_ were tested. Players were divided into forwards, midfielders, and defenders. Forwards had significantly (*p* = 0.012) higher relative VO_2max_ than defenders (59.8 ± 4.3 compared to 53.0 ± 5.6 mL/kg/min). No significant differences for any of the other measurements were observed between positions. This is the first study to present the physical characteristics between playing positions in off- and on-ice tests for male bandy players competing at the highest level. Today’s bandy players are heavier and have lower relative VO_2max_ compared with players in the early 1990s. However, their work capacities have increased since their absolute VO_2max_ is higher. These results provide benchmark values that can serve as a foundation for strength and conditioning professionals when designing future training programs.

## 1. Introduction

Bandy is a historically popular winter sport in Nordic and Siberian countries. The game is played on an ice surface wearing skates and using a stick, similar to ice hockey, while the size of the playing field, playtime, number of players on the field, and rules are similar to soccer. This high-intensity sport is performed with explosive activities, such as sprinting, turning, and battling for a loose ball. Skating velocities of up to 37 km/h, and distances covered of 23 km have recently been reported during a game [1]. A well-developed anaerobic system to handle this repeated high-intensity muscle work, and to produce high force and power, as well as a high aerobic ability are vital. The physiologic requirements are supported by two recently published studies, one on heart rate measurements and one on time–motion analyses during gameplay [1,2]. Workload and the time spent on the ice vary between positions, but an active playing time of 90 min, of which 20 min is performed at a high intensity (above the lactate threshold), is not unusual [2]. The time–motion analysis displays that defensive players skate at longer total distances during a game compared to offensive players. Offensive players in contrast spend more time at fast velocities and display a higher mean skating velocity than defensive players [1].

The Swedish elite bandy league is considered one of the world’s best leagues with some of the highest ranked players. It is important to supplement the above-mentioned time–motion analysis and heart rate measurements with physical characteristics to obtain a full account of what is required to play at this level. Studies examining elite bandy players are sparse. Previous research has mainly focused on injury description, injury prevalence, and game demands [3,4,5,6,7,8]. Two studies from the early 1990s [9,10] presented information about elite bandy players’ physical characteristics. It is realistic to assume that the physical characteristics of bandy players today are not the same as 30 years ago, especially since this is the case in other team sports, such as ice hockey and soccer. These sports have become more physically demanding over the years and now require athletes that are stronger [11,12], faster [12,13,14], and more endurable [11,15]. The only physical characteristic that has been recently examined in bandy players is the countermovement jump (CMJ) height [16]. However, this measurement cannot be used as a benchmark for elite players because the study only included players competing at a lower national level. The physical characteristic differences of playing positions have not been examined, and no study has measured well-established off- and on-ice tests enabling the comparison of athletic abilities both within and outside the sport of bandy.

To address this gap in the literature, the main objective of this study was to assess anthropometric data and physical characteristics off- and on-ice for elite male bandy players at different playing positions. Better physical characteristic knowledge regarding elite-level bandy players will assist strength and conditioning professionals in the design of training programs to improve performance and reduce the risk of injury.

## 2. Materials and Methods

### 2.1. Experimental Design Overview

A cross-sectional study was conducted examining anthropometric measurements and physical characteristics of Swedish elite male bandy players at the highest national level. A test battery (that included 10 tests) was designed to determine a range of different physiological characteristics, such as aerobic power, muscular strength, jump performance, and on-ice sprint speed. Mean values and variations were determined for each test to provide a descriptive physiological profile of male elite bandy players. Findings from earlier studies performed on bandy players were summarized for comparison.

### 2.2. Participants

Twenty-five male bandy players (26.7 ± 5.0 yr.; 83.8 ± 7.6 kg; 182.3 ± 6.6 cm) participated in the study. They were recruited from three teams playing in the Swedish top elite bandy league, “Elitserien”, and were only included if they had training backgrounds of consistent training at this level for a minimum of two years. The players were divided into forwards, midfielders, and defenders (liberos, defenders, and halves). All participants were familiar with the testing protocols before the initiation of the study, were fully informed about the study, and provided their written informed consent before participating. The study was approved by the Regional Ethical Review Board in Stockholm (Dnr 2018/1357-31/1) and followed the principles outlined in the Declaration of Helsinki.

### 2.3. Procedures

All tests were performed just before the start of the competitive season. The same test leaders (H.P, N.W., and A.O), i.e., three experienced strength and conditioning coaches, conducted the testing sessions. Participants performed the tests on three separate occasions, starting with measurements of body weight (BW) and height, maximal strength, and maximum oxygen uptake (VO_2max_) on the first occasion followed by CMJ, squat jump (SJ), and unilateral and bilateral long jumps on the second occasion, and finally, on-ice sprints on the third occasion. All athletes were advised to avoid any strenuous exercise 48 h before the testing. Each testing session included a standardized moderate intensity warm-up of 10 min, including cycling (before the off-ice tests) and skating (before the on-ice tests). BW measurement using a balance scale (Detecto; Web City, MO, USA) was presented in kilograms with an accuracy of 0.1 kg without shoes and heavy clothes. A stadiometer was used to measure height without shoes to the nearest millimeter.

#### 2.3.1. Aerobic Power

Maximal oxygen uptake was obtained using a graded exercise test (GET) performed to volitional exhaustion on a cycle ergometer (Monark LC6, Vansbro, Sweden) or running on a treadmill (Rodby RL2700E, Vänge, Sweden). The choice of running or cycling was based on which of the two exercises the players were most accustomed to during off-ice training since this has been shown to increase the validity of the test, i.e., to obtain a true VO_2max_ and not a VO_2peak_ [17,18]. Oxygen consumption was measured with Oxycon Pro, an automatic metabolic system (Erich Jaeger GmbH, Hoechberg, Germany), using a mixing chamber for direct gas analysis. The Oxycon Pro automatic metabolic system has proven high validity and reliability [19]. The GET on the cycle ergometer was initiated at 260 W and consisted of 1-min of work stages at a pedaling cadence between 80 and 90 rpm. The workload was increased at 20 W for every stage until volitional exhaustion (when the participant was no longer able to maintain a cadence above 70 rpm). The treadmill GET test was initiated at 14 km/h and consisted of 1-min work stages. The workload was increased from 14 to 17 km/h followed by a 1-degree elevation until volitional exhaustion. A pulse band from Polar Electro Oy (Kempele, Finland) was used to measure heart rate (model RS400). The following criteria had to be met to assure a valid VO_2max_ test: (1) leveling off (plateau) of VO_2max_ despite increased braking force on the bike or elevation on the treadmill, (2) a respiratory exchange ratio (VCO_2_/VO_2_) above 1.10, (3) a heart rate 5 beats from the participant’s maximal heart rate, and (4) a subjective rating of perceived exertion above 17 for both heart and legs on Borg’s RPE scale.

#### 2.3.2. Maximal Strength

Standing position isometric mid-thigh pull (IMTP) strength was measured using a portable single-axial load cell described by James et al. [20]. The analog signal from the load cell (Bofors KRG-4 T10; Nobel Elektronik, Karlskoga, Sweden; range 0–4 kN) was converted to digital (CED2501; CED Limited, Cambridge, UK) and displayed with a sampling frequency of 1500 Hz using a 12-bit resolution. Spike 2 software (Cambridge Electronic design 1988–2012) was used to transfer the data to Microsoft Excel 2016 (Microsoft Corporation, Redmond, WA, USA) where all the analyses were conducted. Each participant was instructed to grab the handle and position his body with the knee angle and hip angle set to 145 degrees. A goniometer was used to measure the joint angle during the test. The subjects were instructed to apply a small amount of pretension before pulling as hard as possible for 3 s once they were placed in the right position. A 3-s countdown initiated isometric action. Two maximal voluntary attempts were performed with 120 s of rest in between. Verbal encouragement was given to all participants for each trial. The recorded result was the highest peak value of the two attempts. This portable IMTP method is a valid and reliable method for assessing peak force [20].

#### 2.3.3. Vertical and Horizontal Jump

CMJ and SJ height were measured and recorded indoors using an optical measurement system (Optojump, Microgate, Bolzano-Bozensh, Italy). The vertical jump height estimation system has been demonstrated to have strong validity and test–retest reliability [21]. The participants used a self-selected jump depth and arm swing during CMJ. SJ was initiated from a 90-degree knee angle measured with a goniometer with arms akimbo. The participants were instructed to fully straighten their knee joints at take-off and try to jump as high as possible in both jumps. The participants had to land with a forefoot strike on landing. A total of three jumps were performed, and each effort was followed by a 60-s passive recovery. A total of six jumps were performed; three jumps for each test (CMJ and SJ), and the highest jump from each test was recorded as the result.

The unilateral and bilateral standing long jumps were performed with toes behind the start line. The participants were instructed to jump forward as far as possible and to take off from one leg during the unilateral long jump and from two legs during the bilateral jump. During both types of jumps, the participants were instructed to land on both feet and maintain balance. The participants were given three attempts for the bilateral long jump and three attempts for each leg for the unilateral long jump. A special synthetic mat was used to ensure that the floor did not become slippery. Each jump was initiated by a countermovement, and the distance was measured from the starting line to the nearest heel. A 60-s rest period separated the jumps, and the best-recorded jump for each test was selected for the final analysis.

#### 2.3.4. Sprints On-Ice

The 15-, 30-, and 15-m flying sprints on-ice were performed at the same outdoor bandy field and during equivalent external environmental conditions, i.e., similar temperature, wind, and ice grooming conditions. A Brower electronic timing system (Draper, UT, USA) with single-beamed photocells was used to collect the results. The timing gates were placed 1 m above the ground, 15 and 30 m from the starting gates. Participants were instructed to start in standing positions, 30 cm behind the first timing gate according to Altmann et al., 2015 [22]. Each participant was given three attempts with a 120-s rest in between. The best attempt was recorded as the result.

### 2.4. Statistical Analysis

The data set was checked for the normal distribution using the Shapiro–Wilk test and by inspection of histograms. All statistical analyses were performed using SPSS (Version 27.0, IBM Corporation, New York, NY, USA). Group comparisons were performed using a one-way ANOVA. Multiple comparisons were performed using the Tukey HSD post hoc test to determine which mean differed significantly in case of significant comparisons between groups. The significant level was set at *p* ≤ 0.05. Data are presented as mean ± standard deviation (SD).

## 3. Results

### 3.1. Physical Characteristics

Participant characteristics are presented in Table 1, Table 2 and Table 3. Injuries and personal reasons prohibited some players from performing the SJ, bilateral long jump, unilateral long jumps, and the on-ice sprints (see Table 2 and Table 3 for details).

### 3.2. Positional Differences

There was a significant difference in relative VO_2max_ between the groups (*p* = 0.015). Forwards had a 12.9% higher VO_2max_ compared with defenders (*p* = 0.012, Table 3). There was no significant positional difference for any of the other measurements.

## 4. Discussion

This is the first study presenting both aerobic (VO_2max_) and anaerobic (sprint, jump, and strength) characteristics, as well as anthropometrics for elite male bandy players. This is also the first study examining the physical characteristics of different playing positions. Forwards, midfielders, and defenders have similar physical characteristics except for VO_2max_, which is higher for forwards. Today’s players are heavier and have lower relative VO_2max_ in comparison to previous studies performed ~30 years ago. However, their work capacities have improved.

Physical characteristics in elite-level bandy players [9,10] from the early 1990s were measured in two previous studies. However, these studies focused on aerobic capacity and did not measure sprint and jump performances. Participants included in these studies had an average BW between 70 and 71 kg and relative maximal oxygen consumption between 60 and 63 mL/kg/min. Our findings show that today’s elite male bandy players are approximately 13 kg heavier and have a relative VO_2max_ that is approximately 10% lower. However, their absolute VO_2max_ is higher (~4.7 L/min versus ~4.3 L/min) and the work capacity of elite-level bandy payers has therefore increased over the last 30 years. Bandy players today have similar BWs as European elite-level ice hockey players (84 vs. 86 kg, respectively) [23]. In comparison to players in the National Hockey League (NHL), bandy players are much lighter (84 compared to 92 kg) [24]. Ice hockey players have not experienced the same drop in relative VO_2max_ [11,15] and today’s NHL players have a VO_2max_ of approximately 58 mL/kg/min [25]. The absolute VO_2max_ and work capacity of ice hockey players are therefore higher compared with bandy players (~5.3 L/min versus 4.7 L/min). This difference could partly be explained by the nature of the games. Bandy is a less intermittent sport compared to ice hockey and does not include the same amount of high-intensity actions that are known to stress/develop the aerobic system. Moreover, even though both sports have become more sprint- and power-oriented, ice hockey has likely experienced an even greater development in this direction. Compared to professional soccer, a sport that is similar to bandy in terms of field size, rules, and playing time, bandy players are heavier (84 kg versus 77 kg) and have a lower relative VO_2max_ (56 versus 59–63 mL/kg/min) [26]. This indicates that bandy is a less aerobic-demanding sport than soccer, which is confirmed by in-game measurements, showing that the relative workload during gameplay is lower for bandy [2] compared with soccer [27]. This is probably the result of bandy players performing less eccentric and vertical movements and maintaining speed while gliding on their skates. This makes bandy less of a weight-bearing sport compared to soccer and, therefore, a high absolute VO_2max_ is more important in bandy.

There was a difference in the present study between positions regarding relative VO_2max_, with forwards displaying a higher relative VO_2max_ compared to the defenders. Forwards perform more high-velocity actions [1] and spend more time above the lactate threshold [2] compared with defenders during a game, while defenders spend more time on the ice and cover a longer total distance [1]. This probably explains the observed difference in VO_2max_ because it is well established that intensity is a more important driver for cardiorespiratory fitness than duration and distance [28]. The gameplay of forwards is probably more similar to the intermittent gameplay of ice hockey.

The result from the present study shows that Swedish elite bandy players jump higher than Norwegian elite bandy players (47 versus 40 cm) [16]. However, this could probably be explained by differences in standardization. In the present study, the participants used arm swings during the CMJ jumps, while this was not allowed in the other study, and an arm swing has been shown to increase jump height by up to 38% [29]. In two recently published studies, elite ice hockey players were tested for CMJ height (with arm swing), 30 m sprint time-on-ice, and 50 feet (15.24 m) flying sprint [30,31]. The results from these studies show that ice hockey and bandy players perform similarly with regard to CMJ height, but that ice hockey players perform considerably better when it comes to 30 m on-ice sprinting (4.07 versus 4.23 s) [31] and 15 m flying sprints (1.57 s compared to 1.72 s) [30]. A potential reason for this discrepancy in sprinting performance might be that ice hockey players have a higher horizontal power-generating capacity compared to bandy players. The standing long jump results from the present study support this assumption because bandy players jumped shorter compared to ice hockey players (248 versus 265 cm) [24]. Another potential explanation is related to the equipment and the testing conditions. The design of the skates defers slightly between ice hockey and bandy. A bandy skate is mainly designed for top speed and gliding while an ice hockey skate is designed for acceleration and start/stop actions. The bandy sprints in the present study were performed outdoors while the ice hockey sprints were performed indoors; therefore, differences in wind and ice grooming conditions could have influenced the results.

Despite the practical novelty of the present findings, this study is not without limitations. Categorization between playing positions proved challenging because some players shifted between positions, and some players played in multiple positions during the season. However, it was clear what positions they had played most of the time during the season and should have been categorized into. IMTP was performed without lifting straps; therefore, it cannot be excluded that grip strength was a limiting factor during the test (this needs to be considered when interpreting the results). Although the total number of participants was relatively high for a study examining elite athletes, the number of participants in each subgroup was low. Therefore, the subgroup analysis should be interpreted with caution. There is a clear need for more research within the field of bandy, especially on women and youth, but also on the relationship between physical capacity and on-ice playing performance.

## 5. Conclusions

This is the first study on elite male bandy players presenting both aerobic (VO_2max_) and anaerobic (sprint, jump, and strength) characteristics, as well as anthropometric measurements. Bandy forwards, midfielders, and defenders have similar physical characteristics, except for VO_2max_, which is higher for forwards. Therefore, a similar training strategy can be applied for all positions regarding strength, speed, and power, but not for endurance. Today’s bandy players are heavier and have lower relative VO_2max_ compared with studies performed ~30 years ago. However, their work capacities (absolute VO_2max_) have improved. The results from the present study can be used as benchmark values for strength and conditioning professionals when evaluating the strengths and weaknesses of bandy players and designing training programs.

## Figures and Tables

**Table 1:** Anthropometric characteristics of the participants.

Variable	Mean (SD) (All Groups)	Position	Mean (SD)	Other Studies Including Bandy Athletes
Body mass (kg) (n = 25)	83.8 ± 7.6	Forwards (n = 7) Midfielders (n = 8) Defenders (n = 10)	83.1 ± 7.8 80.9 ± 6.3 86.5 ± 8.3	*Hakkinen et al. [9]:* Elite male bandy players from Finland (n = 10), 70.3 ± 4.8. *Hakkinen et al. [10]:* Elite male bandy players from Finland (n = 9), 71.0 ± 4.4. *Blomqvist et al. [2]:* Elite male bandy players from Sweden (n = 10), 80.6 ± 4.6. *Persson et al. [1]:* Elite male bandy players from Sweden (n = 10), 82.8 ± 4.2
Height (cm) (n = 25)	182.4 ± 6.6	Forwards (n = 7) Midfielders (n = 8)	182.9 ± 7.6 180.7 ± 5.3	*Hakkinen et al. [9]:* Elite male bandy players from Finland (n = 10), 175.4 ± 4.5
		Defenders (n = 10)	183.4 ± 7.2	*Hakkinen et al. [10]:* Elite male bandy players from Finland (n = 9), 175.1 ± 4.7.
				*Blomqvist et al. [2]:* Elite male bandy players from Sweden (n = 10), 179.6 ± 4.2.
				*Persson et al. [1]:* Elite male bandy players from Sweden (n = 10), 180.6 ± 4.4.

Values are presented as mean ± SD.

**Table 2:** Strength and jump characteristics of the participants.

Variable	Mean (SD) (All Groups)	Position	Mean (SD)	Other Studies Including Bandy Athletes
Isometric Mid-Thigh Pull (kg)	195.2 ± 34.5	Forwards (n = 7)	192.1 ± 30.9	
(n = 25)		Midfielders (n = 8)	194.0 ± 32.4	
		Defenders (n = 10)	198.5 ± 41.3	
Squat jump (cm)	36.3 ± 2.7	Forwards (n = 6)	36.6 ± 2.3	
(n = 23)		Midfielders (n = 7)	36.0 ± 2.4	
		Defenders (n = 10)	36.3 ± 3.3	
Countermovement jump (cm)	47.5 ± 4.5	Forwards (n = 7)	47.6 ± 3.8	*Haugen et al. [16]:* Norwegian national-team bandy players (n = 73), 39.5 ± 5.0.
(n = 25)		Midfielders (n = 8)	46.3 ± 4.2	
		Defenders (n = 10)	48.4 ± 5.2	
Long jump bilateral (cm)	248.3 ± 14.8	Forwards (n = 7)	251.4 ± 19.3	
(n = 24)		Midfielders (n = 7)	245.2 ± 12.6	
		Defenders (n = 10)	248.4 ± 13.8	
Long jump unilateral left (cm)	211.9 ± 8.5	Forwards (n = 7)	213.4 ± 6.1	
(n = 21)		Midfielders (n = 6)	208.1 ± 10.8	
		Defenders (n = 8)	213.2 ± 8.5	
Long jump unilateral right (cm)	210.3 ± 11.6	Forwards (n = 7)	213.1 ± 15.1	
(n = 21)		Midfielders (n = 6)	205.8 ± 6.2	
		Defenders (n = 8)	211.2 ± 11.6	

Values are presented as mean ± SD.

**Table 3:** Endurance and sprint characteristics of the participants.

Variable	Mean (SD) (All Groups)	Position	Mean (SD)	Other Studies Including Bandy Athletes
VO_2 max_ (mL/kg/min)	56.0 ± 5.1	Forwards (n = 7)	59.8 ± 4.3 *	*Hakkinen et al. [9]:* Elite male bandy players from Finland (n = 10), 60.0 ± 4.8.
(n = 25)		Midfielders (n = 8)	56.6 ± 2.1	
		Defenders (n = 10)	53.0 ± 5.6	*Hakkinen et al. [10]:* Elite male bandy players from Finland (n = 9), 63.2 ± 6.0.
VO_2max_ (L/min)	4.7 ± 0.5	Forwards (n = 7)	4.9 ± 0.4	*Hakkinen et al. [9]:* Elite male bandy players from Finland (n = 10), 4.2 ± 0.3
(n = 25)		Midfielders (n = 8)	4.6 ± 0.4	
		Defenders (n = 10)	4.6 ± 0.6	*Hakkinen et al. [10]:* Elite male bandy players from Finland (n = 9), 4.5 ± 0.5.
On-ice sprint 15 m (s)	2.50 ± 0.09	Forwards (n = 7)	2.54 ± 0.09	
(n = 22)		Midfielders (n = 6)	2.52 ± 0.07	
		Defender (n = 9)	2.47 ± 0.09	
On-ice sprint 30 m (s)	4.23 ± 0.12	Forwards (n = 7)	4.27 ± 0.13	
(n = 22)		Midfielders (n = 6)	4.24 ± 0.10	
		Defender (n = 9)	4.19 ± 0.12	
On-ice sprint 15 m flying (s)	1.72 ± 0.05	Forwards (n = 7) Midfielders (n = 6)	1.73 ± 0.04 1.73 ± 0.06	
(n = 22)		Defender (n = 9)	1.72 ± 0.06	

Values are presented as mean ± SD. *, *p* < 0.05 vs. defenders.

## Data Availability

All data generated and analyzed during this study are included in this study.

## References

[1] Persson E., Andersson M., Blomqvist S. (2021). Differences in Physical Demands Among Offensive and Defensive Players in Elite Men Bandy. Res. Q. Exerc. Sport.

[2] Blomqvist S., Ervasti P.E., Elcadi G.H. (2018). Evaluating Physical Workload by Position During Match in Elite Bandy. J. Strength Cond. Res..

[3] Sane J. (1988). Comparison of maxillofacial and dental injuries in four contact team sports: American football, bandy, basketball, and handball. Am. J. Sports Med..

[4] Paajanen H., Hermunen H., Karonen J. (2011). Effect of heavy training in contact sports on MRI findings in the pubic region of asymptomatic competitive athletes compared with non-athlete controls. Skelet. Radiol..

[5] Risto O., Timpka T. (2007). Towards safe environments for youth sports: Impact of a fair play programme on injury rates in youth bandy. Int. J. Inj. Control Saf. Promot..

[6] Timpka T., Risto O., Lindqvist K. (2002). Injuries in competitive youth bandy: An epidemiological study of a league season. Med. Sci. Sports Exerc..

[7] Sandmark H., Vingard E. (1999). Sports and risk for severe osteoarthrosis of the knee. Scand. J. Med. Sci. Sports.

[8] Johansson M., Ervasti P.E., Blomqvist S. (2021). An Analysis of Acceleration, Deceleration and High-Intensity Skating during Elite Bandy Match-Play: A Case Study. Sports.

[9] Hakkinen K., Sinnemaki P. (1990). Maximum oxygen uptake, anaerobic power and neuromuscular performance characteristics in bandy players of two different competitive levels. J. Sports Med. Phys. Fit..

[10] Hakkinen K., Sinnemäki P. (1991). Changes in physical fitness profile during the competitive season in elite bandy players. J. Sports Med. Phys. Fit..

[11] Quinney H.A., Dewart R., Game A., Snydmiller G., Warburton D., Bell G. (2008). A 26 year physiological description of a National Hockey League team. Appl. Physiol. Nutr. Metab..

[12] Secora C.A., Latin R.W., Berg K.E., Noble J.M. (2004). Comparison of physical and performance characteristics of NCAA Division I football players: 1987 and 2000. J. Strength Cond. Res..

[13] Haugen T.A., Tønnessen E., Seiler S. (2012). Speed and countermovement-jump characteristics of elite female soccer players, 1995–2010. Int. J. Sports Physiol. Perform..

[14] Haugen T.A., Tonnessen E., Seiler S. (2013). Anaerobic performance testing of professional soccer players 1995–2010. Int. J. Sports Physiol. Perform..

[15] Montgomery D.L. (2006). Physiological profile of professional hockey players—A longitudinal comparison. Appl. Physiol. Nutr. Metab..

[16] Haugen T.A., Breitschädel F., Wiig H., Seiler S. (2020). Countermovement Jump Height in National-Team Athletes of Various Sports: A Framework for Practitioners and Scientists. Int. J. Sports Physiol. Perform..

[17] Caputo F., Denadai B.S. (2004). Effects of aerobic endurance training status and specificity on oxygen uptake kinetics during maximal exercise. Eur. J. Appl. Physiol..

[18] Costa M.M., Russo A.K., Pićarro I.C., Barros Neto T.L., Silva A.C., Tarasantchi J. (1989). Oxygen consumption and ventilation during constant-load exercise in runners and cyclists. J. Sports Med. Phys. Fit..

[19] Foss O., Hallen J. (2005). Validity and stability of a computerized metabolic system with mixing chamber. Int. J. Sports Med..

[20] James L.P., Roberts L.A., Haff G.G., Kelly V.G., Beckman E.M. (2017). Validity and Reliability of a Portable Isometric Mid-Thigh Clean Pull. J. Strength Cond. Res..

[21] Glatthorn J.F., Gouge S., Nussbaumer S., Stauffacher S., Impellizzeri F.M., Maffiuletti N.A. (2011). Validity and reliability of Optojump photoelectric cells for estimating vertical jump height. J. Strength Cond. Res..

[22] Altmann S., Hoffmann M., Kurz G., Neumann R., Woll A., Haertel S. (2015). Different Starting Distances Affect 5-m Sprint Times. J. Strength Cond. Res..

[23] Vigh-Larsen J.F., Beck J.H., Daasbjerg A., Knudsen C.B., Kvorning T., Overgaard K., Andersen T.B., Mohr M. (2019). Fitness Characteristics of Elite and Subelite Male Ice Hockey Players: A Cross-Sectional Study. J. Strength Cond. Res..

[24] Boucher V.G., Parent A.A., St-Jean Miron F., Leone M., Comtois A.S. (2020). Comparison Between Power Off-Ice Test and Performance On-Ice Anaerobic Testing. J. Strength Cond. Res..

[25] Delisle-Houde P., Chiarlitti N.A., Reid R.E.R., Andersen R.E. (2019). Predicting On-Ice Skating Using Laboratory- and Field-Based Assessments in College Ice Hockey Players. Int. J. Sports Physiol. Perform..

[26] Slimani M., Nikolaidis P.T. (2019). Anthropometric and physiological characteristics of male soccer players according to their competitive level, playing position and age group: A systematic review. J. Sports Med. Phys. Fit..

[27] Rebelo A., Brito J., Seabra A., Oliveira J., Drust B., Krustrup P. (2012). A new tool to measure training load in soccer training and match play. Int. J. Sports Med..

[28] Laursen P.B., Jenkins D.G. (2002). The scientific basis for high-intensity interval training: Optimising training programmes and maximising performance in highly trained endurance athletes. Sports Med..

[29] Vaverka F., Jandačka D., Zahradník D., Uchytil J., Farana R., Supej M., Vodičar J. (2016). Effect of an Arm Swing on Countermovement Vertical Jump Performance in Elite Volleyball Players: FINAL. J. Hum. Kinet..

[30] Runner A.R., Lehnhard R.A., Butterfield S.A., Tu S., O’Neill T. (2016). Predictors of Speed Using Off-Ice Measures of College Hockey Players. J. Strength Cond. Res..

[31] Vigh-Larsen J.F., Haverinen M.T., Knudsen C.B., Daasbjerg A., Beck J.H., Overgaard K., Mohr M., Andersen T.B. (2021). The relationship between age and fitness profiles in elite male ice hockey players. J. Sports Med. Phys. Fit..

